# Linking Cerebral Malaria Pathogenesis to APOE-Mediated Amyloidosis: Observations and Hypothesis

**DOI:** 10.1007/s12035-024-04366-3

**Published:** 2024-07-18

**Authors:** Mwikali Kioko, Shaban Mwangi, James M. Njunge, James A. Berkley, Philip Bejon, Abdirahman I. Abdi

**Affiliations:** 1 Bioscience Department, KEMRI-Wellcome Trust Research Programme, Kilifi, Kenya; 2 Centre for Tropical Medicine and Global Health, Nuffield Department of Medicine, University of Oxford, Oxford, UK; 3 Pwani University Biosciences Research Centre, Pwani University, Kilifi, Kenya

**Keywords:** Cerebral malaria, Alzheimer’s disease, *Plasmodium falciparum*, Amyloidosis, Apolipoprotein E, Amyloid-beta

## Abstract

Although most children with cerebral malaria fully recover, more than a fifth of the survivors develop post-discharge neurodevelopmental sequelae suggestive of advanced neuronal injury. However, the cerebral molecular processes initiating neurological dysfunction in cerebral malaria are still debatable. In this article, we explore available data and hypothesise that cerebral malaria might be linked to APOE-mediated amyloidosis, one of the pathological processes associated with Alzheimer’s disease. If our hypothesis is tested and found to be true, it could have far-reaching implications for what we know about cerebral malaria pathogenesis.

**Supplementary Information:**

The online version contains supplementary material available at 10.1007/s12035-024-04366-3.

## Introduction

Cerebral malaria (CM) is a neurological syndrome caused by *Plasmodium falciparum*, and its mechanism of pathogenesis is tightly linked to the adhesion and sequestration of parasitised erythrocytes in the brain microvasculature [1]. Vascular-related pathological events such as brain endothelial activation and dysfunction [2], infiltration of activated CD8^+^ T cells into the brain microvasculature [3] and disruption of the blood–brain barrier (BBB) [4] are thought to occur secondary to the sequestration of infected erythrocytes. However, since these pathological mechanisms are vascular-related, the exact molecular processes occurring in the brain tissues that cause neuronal dysfunction in CM are poorly understood. Although most CM patients fully recover, 15–20% die, and greater than 20% of the survivors develop long-term post-discharge neurodevelopmental sequelae, including hemiplegia, aphasia, cortical blindness and ataxia [5], indicating a similarity between CM and neurodegeneration. Here, we explore available literature and hypothesise that CM might share a common pathological pathway with Alzheimer’s disease (AD) despite the former being largely remediable.

## APOE Modulates Amyloidosis in AD in a Gene Dose-Dependent Manner

AD is a neurological disorder caused by age-associated changes in the brain, alongside environmental and genetic factors. Some age-associated changes that could lead to AD include brain atrophy [6], vascular damage [7, 8], neuroinflammation [9], production of free radicals leading to excessive peroxidation of lipids [10] and dysregulation of energy metabolism in brain cells leading to energy deficits and neuronal dysfunction [11]. Some AD cases are caused by the accumulation and deposition of amyloid-β (Aβ) peptides in the brain, a process termed amyloidosis [12], followed by hyperphosphorylation of tau to form toxic neurofibrillary tangles, a process termed tauopathy [13]. However, some studies also report that tauopathy can occur independently of amyloidosis. The Aβ peptides are derived from the proteolysis of the neuronal amyloid precursor protein (APP) [12]. A cholesterol-transporting protein called apolipoprotein E (APOE), secreted primarily by astrocytes, plays a central role in AD by modulating either the transcription of APP [14] or the clearance of Aβ [15]. APOE and APP levels are increased in the cerebral spinal fluid (CSF) of AD patients [16–18] and in the brain tissues of transgenic mouse models of Aβ amyloidosis [19–22]. In addition, accumulating studies in mouse models of AD suggest that the absence of APOE, either genetically or pharmacologically, dramatically decreases Aβ amyloidosis in a gene dose-dependent manner [19–24]. APOE is expressed in three isoforms, APOE2, APOE3 and APOE4, at a single gene locus, with APOE4 being the leading genetic risk factor for AD [25] and the one associated with the highest levels of Aβ synthesis and deposition [14, 15]. Although the APOE4 variant is widely believed to contribute to neurodegeneration by promoting the accumulation of APP and Aβ [14, 15, 19–24], APOE4 has also been associated with vascular dysfunction and BBB leakage [7, 8].

## Amyloidosis-Related CSF Proteins are Associated with CM

**Fig. 1:**
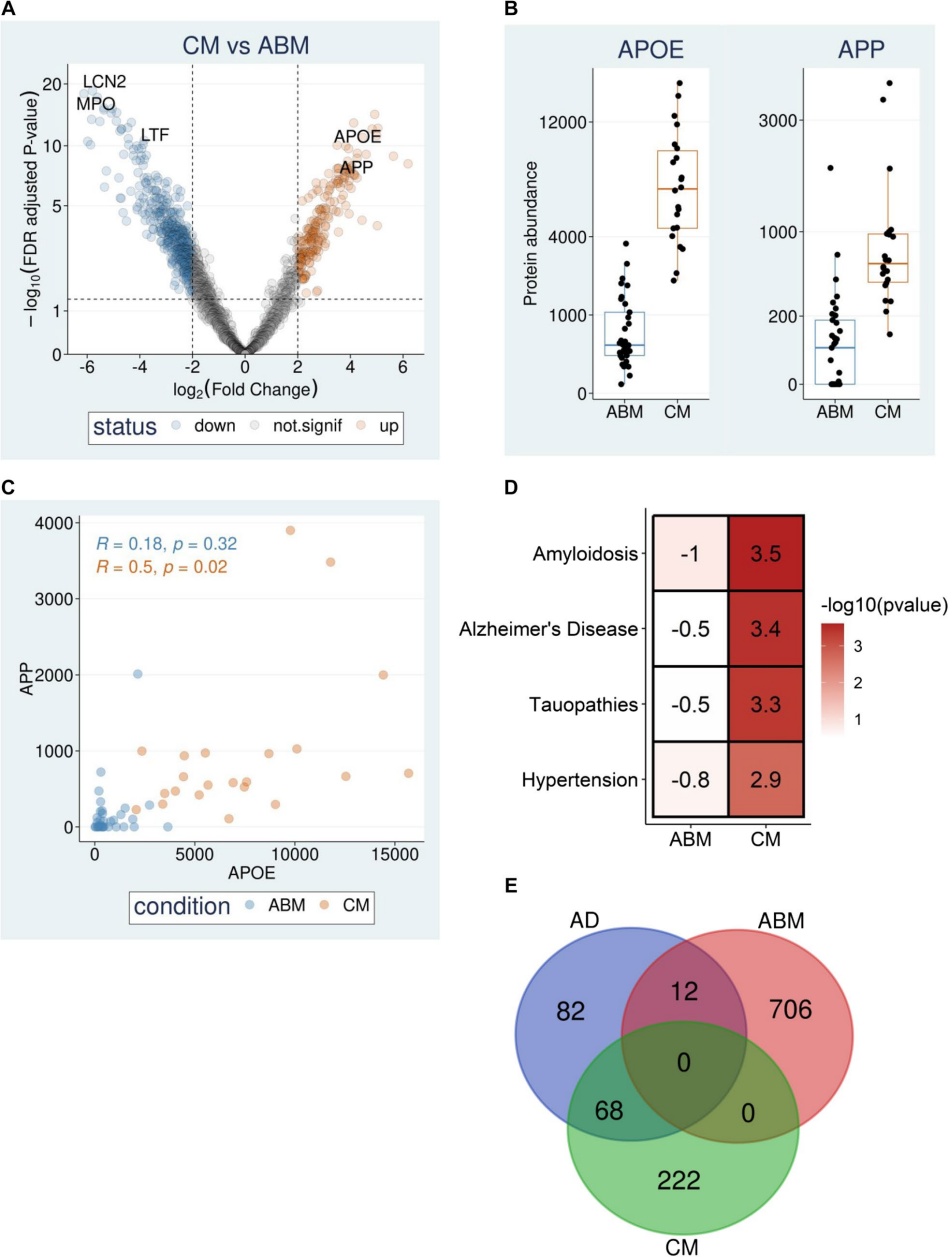
The cerebral spinal fluid (CSF) levels of amyloid-associated proteins are co-increased in cerebral malaria. **A** Volcano plot showing that the CSF protein levels of apolipoprotein E (APOE) and amyloid precursor protein (APP) are elevated in cerebral malaria (CM) compared to acute bacterial meningitis (ABM) at log_2_Fold-Change > 2 and false discovery rate (FDR)-adjusted *p*-value < 0.05. Spectrum searches were performed using Maxquant version 2.0.3, and moderated t.test in limma R package was used to perform the statistical analysis. The colours depict the following: blue, proteins downregulated in CM; grey, not significantly altered proteins; and red, proteins upregulated in CM. Consistent with what was observed previously [33], lipocalin 2 (LCN2), myeloperoxidase (MPO) and lactotransferrin (LTF) have lower CSF protein levels in CM compared to ABM. **B** Boxplots comparing the CSF protein abundance of APOE and APP between CM (*n* = 22) and ABM (*n* = 33). The centre lines show the medians; limits show the median ± interquartile range; whiskers show values 1.5 times above or below the 75th and 25th percentiles, respectively; and each point represents a sample. **C** Scatterplot showing that CSF protein levels of APOE (x-axis) and APP (y-axis) were significantly positively correlated in CM but not in ABM. The Spearman’s rank correlation test was used to calculate the correlation statistics. **D** Enrichment analysis [34] showing the top four disease ontology terms associated with CM compared to ABM. Colour depicts − log10 (*p*-value) while numbers show the enrichment score. **E** Overlap of proteins enriched in CM and ABM [33] with AD-altered proteins from a previous meta-analysis study [35]

The role of APOE in CM pathogenesis has been considered [26–28]. An observational study reported that children (< 5 years of age) with APOE4 isoforms have a higher risk of developing and dying from CM compared to those with other isoforms [28]. Additionally, a recent study observed that APOE^−/−^ mice did not develop experimental CM (ECM), even at 70–80% peripheral parasitemia, had lower parasite sequestration in the brain, reduced disruption of the BBB and decreased infiltration of T cells into the brain [27]. Another mouse study used immunochemistry to show that Aβ accumulates in the brain tissues of ECM-sensitive mice infected with *P. berghei* but not in ECM-resistant mice [29]. The APP protein was upregulated in the brain sections of patients who died from CM relative to those with no clinical cerebral pathology [30]. Also, increased CSF levels of tau were associated with long-term neurological and cognitive deficits in CM patients [31], and anti-tau immunotherapy prevented parasite-induced cognitive impairment and was associated with significantly reduced neuroinflammation and vascular congestion in ECM [32]. While primarily searching for biomarkers of acute bacterial meningitis (ABM) relative to CM, Njunge and colleagues used mass spectrometry to compare the CSF protein profiles of paediatric admissions with either of the two infections [33]. They observed that APOE was among the most upregulated CSF proteins in CM [33]. We reanalysed Njunge et al. proteomic data [33] and observed that in addition to APOE, the CSF levels of APP and other AD-related proteins such as NPTX1, PRNP, NCAM1, SPARC, AGT and IGF2 were also significantly elevated in CM compared to ABM (Fig. 1A, B; Supplementary File Table 1). We also noticed that APOE was significantly positively correlated to APP in CM (*R* = 0.5, *p* = 0.02) but not in ABM (*R* = 0.18, *p* = 0.32) (Fig. 1C), which was concordant with the gene dose-dependent amyloidogenic effect of APOE observed previously in AD [19–24]. When we performed disease ontology enrichment analysis [34], we found that genes upregulated in CM were strongly associated with AD-related terms such as “amyloidosis”, “Alzheimer’s disease” and “tauopathies” (Fig. 1D). Next, we obtained a list of 162 CSF proteins reported in at least two studies to be significantly altered in AD patients compared to healthy controls [35] and overlapped them with proteins that we found significantly enriched in CSF from CM patients compared to ABM (Supplementary File Table 2). This analysis showed that 68 of the 168 AD-altered CSF proteins, including APOE and APP, overlapped with those enriched in CM compared to ABM, while only 12 overlapped with those enriched in ABM (Fig. 1E, Supplementary File Table 2). These observations and data suggest that AD-linked proteins are strongly associated with CM.

## Concluding Remarks

**Fig. 2:**
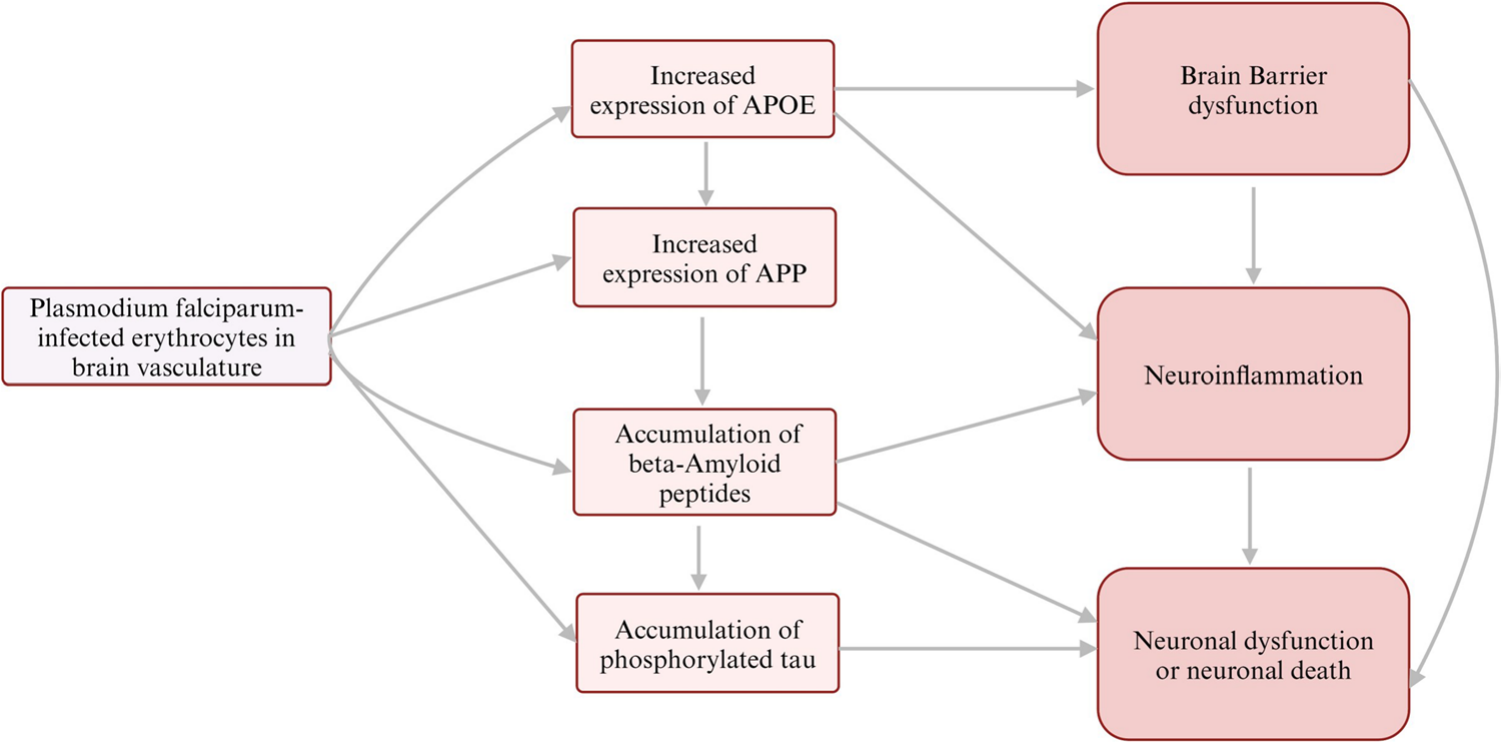
The APOE-APP hypothesis of cerebral malaria. Parasite sequestration in the brain capillaries can trigger the upregulation of APOE and APP, accumulation of amyloid-beta peptides and phosphorylation of neuronal tau. The APOE4 isoform can cause alteration of the blood–brain barrier (BBB) and neuroinflammation. Similarly, brain hypoperfusion may trigger dysregulated vasodilation and breakage of BBB, leading to leakage of vascular material into the brain parenchyma, resulting in further neuroinflammation and dysfunction of neurons. Therapeutic targets that block APOE or prevent the build-up of beta-amyloid peptides and phosphorylated tau could be explored as interventions for post-developmental sequelae observed in cerebral malaria survivors

We have hypothesised and provided partial evidence that druggable AD signatures are augmented in CM. This information might benefit the search for treatment and management of both AD and CM. For instance, amyloidosis-targeted and anti-APOE therapies could be explored to prevent post-infection neurodevelopmental sequelae observed in some children after CM recovery [5] (Fig. 2). Furthermore, the general reversibility of CM might provide insights into how to reverse or prevent the progression of AD. However, additional scientific data will be required to support the amyloidosis hypothesis of CM pathogenesis. First, a multicentre large CSF proteomic study would be required to confirm the co-upregulation of APOE and APP in CM patients relative to age-matched patients with non-plasmodial encephalopathies and healthy community controls. In addition to proteomics, we propose an integrated analysis of the CSF transcriptomes, including mRNA, lncRNA and miRNA [36], to interrogate the similarities between CM and AD further. In vivo amyloid imaging using positron emission tomography could confirm or exclude the accumulation of Aβ and tau in antemortem cases of CM. A large human study to confirm whether the APOE4 isoform is associated with CM would also be highly informative. In summary, we hypothesise that amyloidosis might be a common pathophysiological process underlying the neurological disorders of CM and AD, most likely initiated by parasite-induced co-upregulation of APOE and APP in the cerebral tissues.

## Supplementary Information

Below is the link to the electronic supplementary material.

Supplementary file1 (XLSX 189 KB)

## Data Availability

The proteomic data reanalysed in this short communication was downloaded from the MassIVE repository under the accession number MSV000080979.

## References

[1] Marsh K, Forster D, Waruiru C, Mwangi I, Winstanley M, Marsh V et al (1995) Indicators of life-threatening malaria in African children. N Engl J Med 332(21):1399–404. 10.1056/nejm199505253322102 7723795 10.1056/NEJM199505253322102

[2] Conroy AL, Phiri H, Hawkes M, Glover S, Mallewa M, Seydel KB et al (2010) Endothelium-based biomarkers are associated with cerebral malaria in Malawian children: a retrospective case-control study. PLoS One 5(12):e15291. 10.1371/journal.pone.0015291 21209923 10.1371/journal.pone.0015291 PMC3012131

[3] Riggle BA, Manglani M, Maric D, Johnson KR, Lee M-H, Neto OLA et al (2020) CD8+ T cells target cerebrovasculature in children with cerebral malaria. J Clin Investig 130(3):1128–1138 31821175 10.1172/JCI133474 PMC7269583

[4] Brown H, Hien TT, Day N, Mai NT, Chuong LV, Chau TT et al (1999) Evidence of blood-brain barrier dysfunction in human cerebral malaria. Neuropathol Appl Neurobiol 25(4):331–40. 10.1046/j.1365-2990.1999.00188.x 10476050 10.1046/j.1365-2990.1999.00188.x

[5] Idro R, Carter JA, Fegan G, Neville BG, Newton CR (2006) Risk factors for persisting neurological and cognitive impairments following cerebral malaria. Arch Dis Child 91(2):142–8. 10.1136/adc.2005.077784 16326798 10.1136/adc.2005.077784 PMC2082712

[6] Jack CR Jr, Petersen RC, Xu Y, O’Brien PC, Smith GE, Ivnik RJ et al (1998) Rate of medial temporal lobe atrophy in typical aging and Alzheimer’s disease. Neurology 51(4):993–9. 10.1212/wnl.51.4.993 9781519 10.1212/wnl.51.4.993 PMC2768817

[7] Montagne A, Nation DA, Sagare AP, Barisano G, Sweeney MD, Chakhoyan A et al (2020) APOE4 leads to blood-brain barrier dysfunction predicting cognitive decline. Nature 581(7806):71–6. 10.1038/s41586-020-2247-3 32376954 10.1038/s41586-020-2247-3 PMC7250000

[8] Montagne A, Nikolakopoulou AM, Huuskonen MT, Sagare AP, Lawson EJ, Lazic D et al (2021) APOE4 accelerates advanced-stage vascular and neurodegenerative disorder in old Alzheimer’s mice via cyclophilin A independently of amyloid-β. Nat Aging 1(6):506–20. 10.1038/s43587-021-00073-z 35291561 10.1038/s43587-021-00073-z PMC8920485

[9] Bamberger ME, Harris ME, McDonald DR, Husemann J, Landreth GE (2003) A cell surface receptor complex for fibrillar beta-amyloid mediates microglial activation. J Neurosci 23(7):2665–74. 10.1523/jneurosci.23-07-02665.2003 12684452 10.1523/JNEUROSCI.23-07-02665.2003 PMC6742111

[10] Retz W, Gsell W, Münch G, Rösler M, Riederer P (1998) Free radicals in Alzheimer’s disease. J Neural Transm Suppl 54:221–36. 10.1007/978-3-7091-7508-8_22 9850931 10.1007/978-3-7091-7508-8_22

[11] Ryu W-I, Bormann MK, Shen M, Kim D, Forester B, Park Y et al (2021) Brain cells derived from Alzheimer’s disease patients have multiple specific innate abnormalities in energy metabolism. Mol Psychiatry 26(10):5702–5714. 10.1038/s41380-021-01068-3 33863993 10.1038/s41380-021-01068-3 PMC8758493

[12] Cai W, Li L, Sang S, Pan X, Zhong C (2023) Physiological roles of β-amyloid in regulating synaptic function: implications for AD pathophysiology. Neurosci Bull 39(8):1289–1308. 10.1007/s12264-022-00985-9 36443453 10.1007/s12264-022-00985-9 PMC10387033

[13] Oddo S, Caccamo A, Kitazawa M, Tseng BP, LaFerla FM (2003) Amyloid deposition precedes tangle formation in a triple transgenic model of Alzheimer’s disease. Neurobiol Aging 24(8):1063–70. 10.1016/j.neurobiolaging.2003.08.012 14643377 10.1016/j.neurobiolaging.2003.08.012

[14] Huang YA, Zhou B, Wernig M, Südhof TC (2017) ApoE2, ApoE3, and ApoE4 differentially stimulate APP transcription and Aβ secretion. Cell 168(3):427–41.e21. 10.1016/j.cell.2016.12.044 28111074 10.1016/j.cell.2016.12.044 PMC5310835

[15] Castellano JM, Kim J, Stewart FR, Jiang H, DeMattos RB, Patterson BW et al (2011) Human apoE isoforms differentially regulate brain amyloid-β peptide clearance. Sci Transl Med 3(89):89ra57. 10.1126/scitranslmed.3002156 21715678 10.1126/scitranslmed.3002156 PMC3192364

[16] Higginbotham L, Ping L, Dammer EB, Duong DM, Zhou M, Gearing M et al (2020) Integrated proteomics reveals brain-based cerebrospinal fluid biomarkers in asymptomatic and symptomatic Alzheimer’s disease. Sci Adv 6(43):eaaz9360. 10.1126/sciadv.aaz9360 33087358 10.1126/sciadv.aaz9360 PMC7577712

[17] Sasayama D, Hattori K, Yokota Y, Matsumura R, Teraishi T, Yoshida S et al (2020) Increased apolipoprotein E and decreased TNF-α in the cerebrospinal fluid of nondemented APOE-ε4 carriers. Neuropsychopharmacol Rep 40(2):201–205. 10.1002/npr2.12110 32426945 10.1002/npr2.12110 PMC7722685

[18] Nordengen K, Kirsebom B-E, Henjum K, Selnes P, Gísladóttir B, Wettergreen M et al (2019) Glial activation and inflammation along the Alzheimer’s disease continuum. J Neuroinflammation 16(1):46. 10.1186/s12974-019-1399-2 30791945 10.1186/s12974-019-1399-2 PMC6383268

[19] Kuo Y-M, Crawford F, Mullan M, Kokjohn TA, Emmerling MR, Weller RO et al (2000) Elevated Aβ and apolipoprotein E in AβPP transgenic mice and its relationship to amyloid accumulation in Alzheimer’s disease. Mol Med 6(5):430–439. 10.1007/BF03401785 10952022 PMC1949958

[20] Bales KR, Verina T, Dodel RC, Du Y, Altstiel L, Bender M et al (1997) Lack of apolipoprotein E dramatically reduces amyloid beta-peptide deposition. Nat Genet 17(3):263–4. 10.1038/ng1197-263 9354781 10.1038/ng1197-263

[21] Bien-Ly N, Gillespie AK, Walker D, Yoon SY, Huang Y (2012) Reducing human apolipoprotein E levels attenuates age-dependent Aβ accumulation in mutant human amyloid precursor protein transgenic mice. J Neurosci 32(14):4803–11. 10.1523/jneurosci.0033-12.2012 22492035 10.1523/JNEUROSCI.0033-12.2012 PMC3433173

[22] Kim J, Jiang H, Park S, Eltorai AE, Stewart FR, Yoon H et al (2011) Haploinsufficiency of human APOE reduces amyloid deposition in a mouse model of amyloid-β amyloidosis. J Neurosci 31(49):18007–12. 10.1523/jneurosci.3773-11.2011 22159114 10.1523/JNEUROSCI.3773-11.2011 PMC3257514

[23] Kim J, Eltorai AE, Jiang H, Liao F, Verghese PB, Kim J et al (2012) Anti-apoE immunotherapy inhibits amyloid accumulation in a transgenic mouse model of Aβ amyloidosis. J Exp Med 209(12):2149–56. 10.1084/jem.20121274 23129750 10.1084/jem.20121274 PMC3501350

[24] Liao F, Li A, Xiong M, Bien-Ly N, Jiang H, Zhang Y et al (2018) Targeting of nonlipidated, aggregated apoE with antibodies inhibits amyloid accumulation. J Clin Invest 128(5):2144–55. 10.1172/jci96429 29600961 10.1172/JCI96429 PMC5919821

[25] Schmechel DE, Saunders AM, Strittmatter WJ, Crain BJ, Hulette CM, Joo SH et al (1993) Increased amyloid beta-peptide deposition in cerebral cortex as a consequence of apolipoprotein E genotype in late-onset Alzheimer disease. Proc Natl Acad Sci U S A 90(20):9649–53. 10.1073/pnas.90.20.9649 8415756 10.1073/pnas.90.20.9649 PMC47627

[26] Aucan C, Walley AJ, Hill AV (2004) Common apolipoprotein E polymorphisms and risk of clinical malaria in the Gambia. J Med Genet 41(1):21–4. 10.1136/jmg.2003.011981 14729824 10.1136/jmg.2003.011981 PMC1757275

[27] Kassa FA, Van Den Ham K, Rainone A, Fournier S, Boilard E, Olivier M (2016) Absence of apolipoprotein E protects mice from cerebral malaria. Sci Rep 6(1):33615. 10.1038/srep33615 27647324 10.1038/srep33615 PMC5028887

[28] Lima-Cooper G, Ouma BJ, Datta D, Bond C, Soto AA, Conroy AL, et al (2023) Apolipoprotein-E4: risk of severe malaria and mortality and cognitive impairment in pediatric cerebral malaria. Pediatr Res. 10.1038/s41390-023-02912-8 10.1038/s41390-023-02912-8 PMC12009649 38007518

[29] Delahaye NF, Coltel N, Puthier D, Barbier M, Benech P, Joly F et al (2007) Gene expression analysis reveals early changes in several molecular pathways in cerebral malaria-susceptible mice versus cerebral malaria-resistant mice. BMC Genomics 8:452. 10.1186/1471-2164-8-452 18062806 10.1186/1471-2164-8-452 PMC2246131

[30] Medana IM, Day NP, Hien TT, Mai NT, Bethell D, Phu NH et al (2002) Axonal injury in cerebral malaria. Am J Pathol 160(2):655–666. 10.1016/s0002-9440(10)64885-7 11839586 10.1016/S0002-9440(10)64885-7 PMC1850649

[31] Datta D, Conroy AL, Castelluccio PF, Ssenkusu JM, Park GS, Opoka RO et al (2020) Elevated cerebrospinal fluid tau protein concentrations on admission are associated with long-term neurologic and cognitive impairment in Ugandan children with cerebral malaria. Clin Infect Dis 70(6):1161–8. 10.1093/cid/ciz325 31044219 10.1093/cid/ciz325 PMC7319060

[32] AkideNdunge OB, Shikani HJ, Dai M, Freeman BD, Desruisseaux MS (2023) Effects of anti-tau immunotherapy on reactive microgliosis, cerebral endotheliopathy, and cognitive function in an experimental model of cerebral malaria. J Neurochem. 10.1111/jnc.15972 10.1111/jnc.15972 PMC10596299 37814468

[33] Njunge JM, Oyaro IN, Kibinge NK, Rono MK, Kariuki SM, Newton CR et al (2017) Cerebrospinal fluid markers to distinguish bacterial meningitis from cerebral malaria in children. Wellcome Open Res 2:47. 10.12688/wellcomeopenres.11958.2 29181450 10.12688/wellcomeopenres.11958.2 PMC5686508

[34] Febbo PG, Mulligan MG, Slonina DA, Stegmaier K, Di Vizio D, Martinez PR et al (2007) Literature Lab: a method of automated literature interrogation to infer biology from microarray analysis. BMC Genomics 8(1):461. 10.1186/1471-2164-8-461 18088408 10.1186/1471-2164-8-461 PMC2244637

[35] Pedrero-Prieto CM, García-Carpintero S, Frontiñán-Rubio J, Llanos-González E, Aguilera García C, Alcaín FJ et al (2020) A comprehensive systematic review of CSF proteins and peptides that define Alzheimer’s disease. Clin Proteomics 17(1):21. 10.1186/s12014-020-09276-9 32518535 10.1186/s12014-020-09276-9 PMC7273668

[36] Abedpoor N, Taghian F, Hajibabaie F (2022) Cross brain-gut analysis highlighted hub genes and LncRNA networks differentially modified during leucine consumption and endurance exercise in mice with depression-like behaviors. Mol Neurobiol 59(7):4106–23. 10.1007/s12035-022-02835-1 35476290 10.1007/s12035-022-02835-1 PMC9045027

